# Intracoronary Imaging, Cholesterol Efflux, and Transcriptomics after Intensive Statin Treatment in Diabetes

**DOI:** 10.1038/s41598-017-07029-7

**Published:** 2017-08-01

**Authors:** Surbhi Chamaria, Kipp W. Johnson, Yuliya Vengrenyuk, Usman Baber, Khader Shameer, Aparna A. Divaraniya, Benjamin S. Glicksberg, Li Li, Samit Bhatheja, Pedro Moreno, Akiko Maehara, Roxana Mehran, Joel T. Dudley, Jagat Narula, Samin K. Sharma, Annapoorna S. Kini

**Affiliations:** 1 Mount Sinai Heart, Mount Sinai Hospital, New York, USA; 2 Institute for Next Generation Healthcare, Icahn School of Medicine at Mount Sinai, New York, USA; 3 Department of Genetics and Genomic Sciences, Icahn Institute for Genetics and Genomic Sciences, Icahn School of Medicine, New York, USA; 4 Department of Population Health and Health Policy, Icahn School of Medicine at Mount Sinai, New York, USA; 5 Columbia University Medical Center, New York, USA

## Abstract

Residual atherothrombotic risk remains higher in patients with versus without diabetes mellitus (DM) despite statin therapy. The underlying mechanisms are unclear. This is a retrospective post-hoc analysis of the YELLOW II trial, comparing patients with and without DM (non-DM) who received rosuvastatin 40 mg for 8–12 weeks and underwent intracoronary multimodality imaging of an obstructive nonculprit lesion, before and after therapy. In addition, blood samples were drawn to assess cholesterol efflux capacity (CEC) and changes in gene expression in peripheral blood mononuclear cells (PBMC). There was a significant reduction in low density lipoprotein-cholesterol (LDL-C), an increase in CEC and beneficial changes in plaque morphology including increase in fibrous cap thickness and decrease in the prevalence of thin cap fibro-atheroma by optical coherence tomography in DM and non-DM patients. While differential gene expression analysis did not demonstrate differences in PBMC transcriptome between the two groups on the single-gene level, weighted gene coexpression network analysis revealed two modules of coexpressed genes associated with DM, Collagen Module and Platelet Module, related to collagen catabolism and platelet function respectively. Bayesian network analysis revealed key driver genes within these modules. These transcriptomic findings might provide potential mechanisms responsible for the higher cardiovascular risk in DM patients.

## Introduction

Diabetes mellitus (DM) has been recognized as an independent major cardiovascular risk factor since the publication of the first large-scale epidemiologic investigations in the 1970’s^1^. DM, in the absence of previous cardiovascular disease, confers a risk of adverse cardiovascular events similar to that faced by non-diabetic (non-DM) individuals with a history of previous cardiovascular events, thus representing a ‘coronary risk equivalent.’ Accordingly, current guidelines recommend that diabetic patients be treated with statins regardless of their cardiovascular history^2–4^. In a series of pivotal clinical trials between 1989 and 2000, statins have been shown to reduce both atherogenic lipoproteins and cardiovascular morbidity and mortality^5–8^. A post-hoc subgroup analysis of the data on diabetic patients included in the Scandinavian Simvastatin Survival Study provided the first trial-based evidence that lowering cholesterol with simvastatin improves the prognosis of diabetic patients with coronary heart disease (CHD)^9^. Plaque atheroma volume reduction has been demonstrated by intravascular ultrasound (IVUS) in several studies investigating the effects of lipid-lowering therapies, as well as those of anti-hypertensive drugs and anti-diabetic drugs^10–13^. Interestingly, DM appears to be one of the major negative determinants of coronary plaque regression in patients with acute coronary syndrome^14^. Our previous YELLOW II^15^ study (Reduction in Coronary Yellow Plaque, Lipids and Vascular Inflammation by Aggressive Lipid Lowering) was designed to assess the changes in plaque morphology of an obstructive non culprit lesion (NCL) by optical coherence tomography (OCT), near-infrared spectroscopy (NIRS) and IVUS with a comprehensive evaluation of high density lipoprotein (HDL) functionality and peripheral blood mononuclear cells (PBMC) transcriptomics in patients receiving high-dose statin therapy. In this post-hoc analysis we studied the differences in response to statin therapy between DM and non-DM patients and whether the residual cardiovascular risk in patients with stable coronary artery disease (CAD) undergoing percutaneaous coronary intervention (PCI) and concomitant DM could be related to impaired statin-responsiveness.

## Results

Of the 85 patients evaluated in YELLOW II, 37 patients had medically treated diabetes. Among the 37 DM patients, 10 were treated with insulin. We show baseline demographic data in Table 1 and types of antidiabetic medications in Supplementary Table 1. The baseline and follow-up chemical characteristics of the DM and non-DM groups are shown in Table 2. The baseline characteristics were well matched. There were no significant differences in baseline lipid profiles between the two groups. Following intensive statin therapy, there was a significant decrease in LDL-C (P < 0.001) and total cholesterol levels (P < 0.001) in both groups with total cholesterol levels lower in DM group compared to non-DM patients at follow-up (P = 0.045). However, DM group did not demonstrate a significant decrease in hs-CRP levels at follow-up (P = 0.058) or increase in ApoA1 (P = 0.47) in contrast to the non-DM group, which had reduced hs-CRP levels at follow up (P = 0.001) and increased ApoA1 (P = 0.003). Overall levels of hs-CRP (P = 0.034) were lower and ApoA1 levels (P = 0.011) were higher in the non-DM group compared to DM group.

**Table 1:** Baseline Demographics.

	Non Diabetes (n = 48)	Diabetes Mellitus (n = 37)	P value
Age	63.3 ± 12.1	61.5 ± 10.2	0.45
Male	31(65)	27(72)	0.52
Hypertension	42(87)	34(92)	0.43
Hypercholesterolemia	43(89)	32(87)	0.78
Smoking	8(17)	4(10)	0.35
Prior MI	6(13)	6(15)	0.76
Prior PCI	13.4(28)	10(28)	0.10
Baseline Statin Use	40(83)	25(67)	0.09
BMI	28.7 ± 5.3	30.6 ± 4.9	0.09
**Diabetes Medications**			
Oral Hypoglycaemics Only		26	
Insulin Only		3	
Combined		7	
None		1	

Values are mean ± SD or n (%) MI- Myocardial infarction, PCI- Percutaneous coronary intervention, BMI- Body mass index.

**Table 2:** Chemical parameters and cholesterol efflux capacity at baseline and follow up.

	Non Diabetes (n = 48)	Diabetes Mellitus (n = 37)	P Value
**Baseline**			
Total Cholesterol, mg/dl	152.0 ± 42.1	155.1 ± 48.7	0.94
LDL Cholesterol, mg/dl	87.8 ± 37.4	85.5 ± 42.7	0.318
HDL Cholesterol, mg/dl	42.9 ± 12.6	39.3 ± 12.6	0.13
Triglyceride^,^, mg/dl	106.5 ± 59.8	155.0 ± 148.8	0.11
ApoA1, mg/dl	121.4 ± 24.5	118.5 ± 27.1	0.48
hs-CRP, mg/dl	3.2 ± 5.5	3.8 ± 5.9	0.13
HbA1C, % (mmol/mol)	5.9 ± 0.4 (41 ± 4.4)	8.3 ± 2.0 (67 ± 21.9)	<0.001
Cholesterol Efflux Capacity	81.1 ± 0.1	80.4 ± 0.2	0.68
**Follow up**			
Total Cholesterol, mg/dl	119.8 ± 29.9	109.4 ± 29.2	0.045
change	−32.3 ± 39.0	−45.7 ± 45.0	0.11
p value	<0.001	<0.001	
LDL Cholesterol, mg/dl	55.3 ± 28.3	43.4 ± 20.6	0.06
change	−32.5 ± 32.5	−41.1 ± 41.8	0.37
p value	<0.001	<0.001	
HDL Cholesterol, mg/dl	44.5 ± 11.6	41.2 ± 17.7	0.058
change	1.5 ± 8.8	1.9 ± 13.4	0.53
p value	0.261	0.76	
Triglyceride, mg/dl	96.1 ± 49.0	120.6 ± 81.0	0.14
change	−10.4 ± 49.8	−34.4 ± 154.7	0.66
p value	0.18	0.14	
ApoA1, mg/dl	132.1 ± 20.7	120.7 ± 25.0	0.011
change	11.38 ± 24.7	1.6 ± 16.0	0.049
p value	0.003	0.47	
hs-CRP, mg/dl	2.17 ± 3.5	3.23 ± 5.0	0.034
change	−0.9 ± 4.1	−0.5 ± 5.0	0.62
p value	0.001	0.058	
HbA1c	6.0 ± 0.5 (42 ± 5.5)	8.2 ± 1.6 (66 ± 1.6)	<0.001
change	0.04 ± 0.2 (0.4 ± 2.2)	−0.11 ± 1.6 (−1.2 ± 17.5)	0.27
p value	0.34	0.62	
Cholesterol Efflux Capacity	85.3 ± 0.1	83.7 ± 0.2	0.20
change	0.04 ± 0.1	0.03 ± 0.7	0.83
p value	0.007	0.015	

Values are mean ± SD

*LDL denotes low density lipoprotein cholesterol, HDL high density lipoprotein cholesterol, Apo-AI apolipoprotein AI, hs-CRP high-sensitivity C-reactive protein.

### OCT and IVUS/NIRS Imaging Findings

We summarize baseline and follow-up multimodality image findings in Tables 3 and 4. There were no significant differences in OCT findings between the groups at baseline including the minimal fibrous cap thickness (FCT), prevalence of thin cap fibro-atheroma (TCFA), lipid and macrophage accumulation. At follow-up, there was an increase in FCT (DM (P = 0.047) and non-DM (P = 0.001)) and decrease in the prevalence of TCFA (DM (P = 0.025) and non-DM (P = 0.034)) in both groups. There was a significant decrease in lipid arc maximum in DM patients (P = 0.037) and decrease in lipid length in non-DM patients (P = 0.020) at follow up but the total lipid volume index (LVI) was not significantly different between both groups (P = 0.74). Although the DM patients showed a significant reduction in macrophage length (P = 0.011) at follow up, the prevalence of total macrophages (P = 0.035) remained higher in the DM group at follow up. The baseline and follow-up IVUS and NIRS plaque characteristics were not statistically significant, although the total atheroma volume and Lipid core burden index (LCBI) were higher in the DM patients at baseline (Table 4).

**Table 3:** Optical Coherence Tomography findings at baseline and follow up

	Non Diabetes (n = 48)	Diabetes Mellitus (n = 37)	P Value
**Baseline**			
Minimal Lumen Area, mm^2^	1.9 ± 0.8	1.7 ± 0.6	0.48
Lipid Rich Plaque	43(89)	33(90)	0.90
Lipid Arc Maximum,°	146.2 ± 82.9	148.4 ± 77.7	0.69
Lipid Length, mm	5.6 ± 4.3	5.6 ± 4.8	0.93
Lipid Volume Index, ° x mm	648.4 ± 671.9	682.5 ± 673.7	0.81
TCFA*	9(20)	8(21)	0.91
Fibrous cap thickness, μm	98.8 ± 41.8	103.4 ± 42.00	0.52
Macrophages	46(96)	37(100)	0.19
Macrophages arc (max), °	131.7 ± 66.0	141.4 ± 67.8	0.54
Macrophage Length, mm	9.1 ± 5.1	10.7 ± 5.6	0.21
Thrombus	6(13)	8(21)	0.38
Microvessel	35(73)	28(77)	0.71
Calcium deposition	42(87)	33(90)	0.67
Calcium-arc (max), °	123.5 ± 85.2	144.9 ± 80.3	0.17
**Follow up**			
Minimal Lumen Area, mm^2^	1.9 ± 0.8	1.7 ± 0.7	0.46
change	0.05 ± 0.25	−0.00 ± 0.21	0.25
p value	0.22	0.64	
Lipid Rich Plaque	43(89)	31(84)	0.57
p value	>0.99	0.16	
Lipid Arc Maximum, ° x mm	146.4 ± 81.1	130.8 ± 77.7	0.56
change	−0.7 ± 23.2	−16.2 ± 38.8	0.07
p value	0.71	0.037	
Lipid Length, mm	5.16 ± 4.18	4.7 ± 3.7	0.76
change	−0.3 ± −0.8	−0.3 ± 1.2	0.34
p value	0.020	0.52	
Lipid Volume Index, °x mm^**^	576.6 ± 667.7	598.2 ± 563.3	0.74
change	−160.3 ± 387.4	−77.8 ± 366.6	0.17
p value	0.17	0.54	
TCFA^*^	3(7)	3(8)	0.83
p value	0.034	0.025	
Fibrous cap thickness, μm	108.7 ± 39.5	109.1 ± 40.4	0.97
change	12.1 ± 18.6	5.6 ± 14.6	0.19
p value	0.001	0.047	
Macrophages	43(89)	37(100)	0.035
p value	0.18	1.00	
Macrophage arc (max), °	130.7 ± 62.7	127.2 ± 58.7	0.62
Change	−3.6 ± 28.4	−14.3 ± 46.2	0.28
p value	0.63	0.08	
Macrophage Length, mm	8.2 ± 5.2	9.6 ± 5.1	0.30
change	−0.9 ± 2.3	−1.5 ± 2.9	0.62
p value	0.027	0.011	
Thrombus	3(7)	6(16)	0.20
p value	0.32	0.16	
Calcium deposition	42(87)	32(87)	>0.99
p value	>0.99	0.32	
Microvessel	73(35)	82(30.3)	0.35
p value	0.32	0.32	
Calcium-arc (max), °	130.8 ± 83.5	152.8 ± 77.8	0.15
change	3.0 ± 11.2	3.8 ± 12.2	0.78
p value	0.06	0.317	

Values are mean ± SD or n (%) TCFA thin cap fibroatheroma, ^**^Averaged lipid arc x lipid length, *Lipid-rich plaque with the minimal fibrous cap thickness <65 µm.

**Table 4:** IVUS and NIRS findings at baseline and follow up.

	Non Diabetes (n = 48)	Diabetes Mellitus (n = 37)	P Value
**IVUS**			
**Baseline**			
TAV, mm^3^	173.6 ± 96.9	192.7 ± 92.9	0.22
PAV, %	59.7 ± 7.1	61.9 ± 8.0	0.11
Plaque Burden, %	75.5 ± 7.4	76.5 ± 6.8	0.52
**Follow up**			
TAV, mm^3^	172.1 ± 95.6	195.2 ± 96.3	0.31
change	−1.44 ± 13.9	2.5 ± 16.7	0.17
p value	0.74	0.74	
PAV, %	60.1 ± 7.1	62.0 ± 8.0	0.09
change	0.35 ± 2.5	0.15 ± 2.1	0.14
p value	0.27	0.39	
Plaque Burden, %	75.2 ± 7.9	76.5 ± 8.2	0.46
Change	−0.28 ± 5.32	0.005 ± 4.5	0.60
p value	0.987	0.48	
**NIRS**			
**Baseline**			
maxLCBI_4mm_	383.1 ± 162.3	456.1 ± 178.7	0.054
Follow up			
maxLCBI_4mm_	399.6 ± 177.7	400.8 ± 186.0	0.95
change	16.5 ± 124.1	−55.3 ± 181.6	0.034
p value	0.44	0.07	

Values are mean ± SD

IVUS intravascular ultrasound, NIRS near infrared spectroscopy, PAV percent atheroma volume, TAV total atheroma volume, maxLCBI4 mm maximal lipid core burden index in a 4 mm segment.

Finally, we performed a multivariate regression analysis generating several statistical models with the changes in different plaque characteristics as outcomes (Supplemental Table 2). In addition to DM status, we adjusted for baseline statin use, hypertension, smoking, BMI and baseline values for imaging parameters. Diabetes was not found to be an independent predictor of the changes in plaque characteristics after high-dose statin therapy.

### Cholesterol Efflux Capacity (CEC)

Baseline CEC was not different between the two groups (Table 2). At follow-up after high dose statin therapy, CEC significantly increased in both groups; DM (P = 0.015) and non-DM (P = 0.007).

### Differentially Expressed Genes

**Figure 1:**
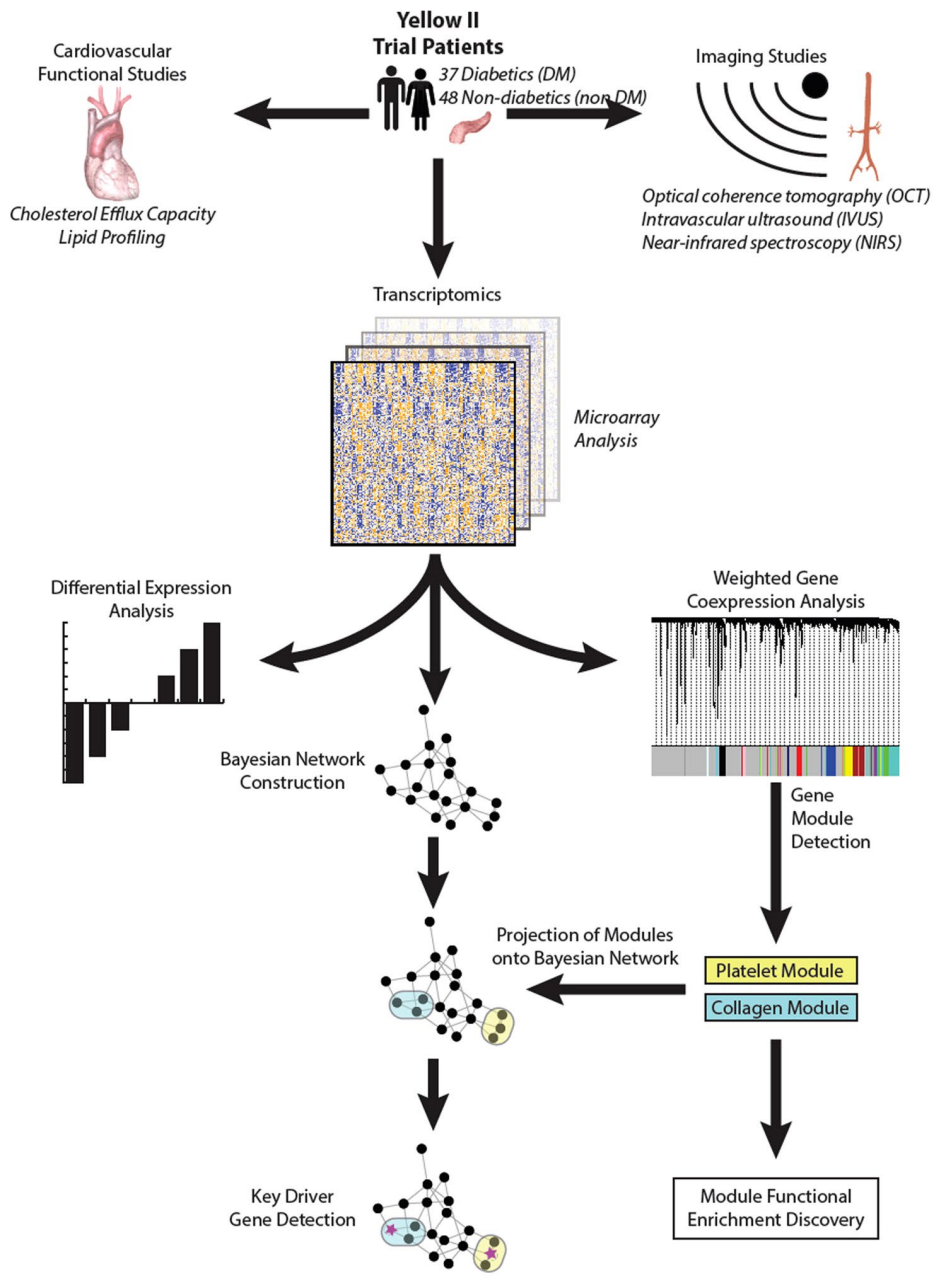
Schematic of transcriptomic analysis workflow.

We mapped the 29,377 microarray probes to 20,819 genes. After normalization, quality control, and the removal of non- and under-expressed genes, 9,981 probes mapped to 9,978 genes (Fig. 1: Schematic of transcriptomic analysis workflow). In the DM group, we did not detect any genes differentially expressed from baseline to follow-up at an FDR-corrected P value of less than 0.05 (Supplementary Figure 1: Differential expression heatmap). In the non-DM group, we detected two genes differentially expressed from baseline to follow-up (*FOLR3 and C15orf54*). *FOLR3*, or folate receptor three (gamma), is a ubiquitously expressed folate receptor that was downregulated following statin exposure. In a previous study, *FOLR3* was upregulated in patients with early onset coronary artery disease compared to matched controls^16^. *C15orf54* is a poorly studied open reading frame on chromosome 15. This locus has previously been found to contain two single nucleotide polymorphisms (SNPs), both associated with cardiovascular phenotypes. The first, rs12907914, was found to be associated with increased risk for heart failure-related, left ventricle hypertrophy in an African American cohort^17^. In an independent study, rs17691453 was found to be associated with the small molecule metabolite dihydroxy docosatrienoic acid, which has been identified as a risk factor for heart failure^18^. Controlling for study time point, we did not detect any genes differentially expressed between DM and non-DM patients.

### Weighted Gene Coexpression Network Analysis

**Figure 2:**
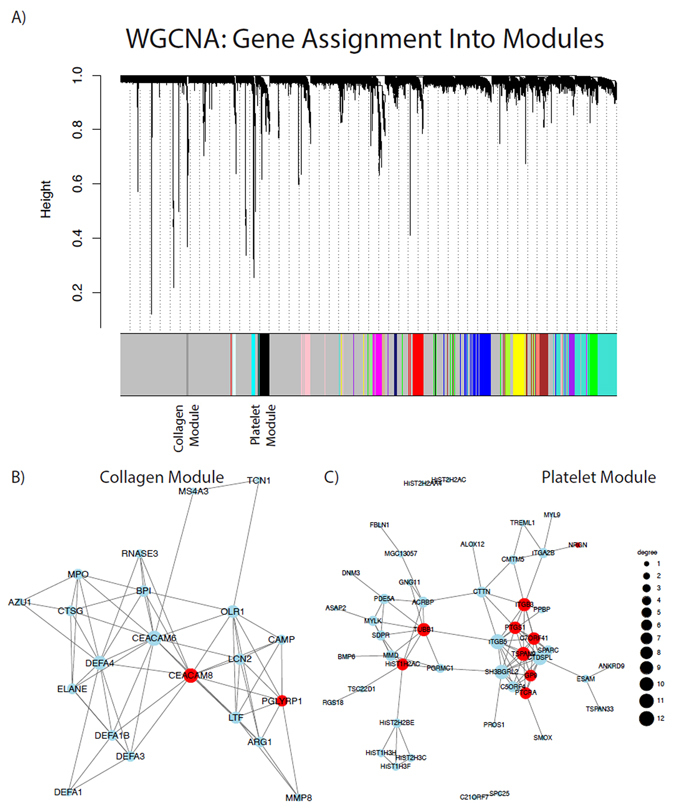
Weighted Gene Coexpression Network Analysis: (WGCNA) (**A**) WGCNA module clustering (**B**) Correlation networks within Collagen Module and (**C**) Platelet Module.

Two of the 19 modules of genes discovered via WGCN analysis were statistically associated with diabetes status (p < 0.10). Significantly associated modules were named for their most significant cardiovascular-associated GO Biological Processes. The first gene expression module (Collagen Module) contained 40 coexpressed genes and the second module (Platelet Module) contained 242 genes (Fig. 2A: WGCNA module clustering. Correlation networks within Collagen Module (2B) and Platelet Module (2 C). In our previous work, we showed the collagen gene module to be associated with fibrous cap thickness independent of DM status^15^. Module summary functions and associated genes are shown in Table 5. The Collagen Module was statistically enriched for 27 GO Biological Processes, 24 GO Cellular Components, 7 GO Molecular Functions, 2 KEGG pathways, 16 CORUM protein complexes, 65 Reactome pathways, 2 Pfam Interpro Domains, 271 ChEA transcription factor (TF) binding sites, 56 ENCODE ChIPseq binding sites, and 66 TRANSFAC and JASPAR TF sites. The Platelet Module was enriched for 73 GO Biological Processes, 15 GO Cellular Components, 7 GO 6 CORUM protein complexes, 10 Reactome pathways, 5 Pfam Interpro Domains, 17 ChEA transcription factor (TF) binding sites, and 1 TRANSFAC, and JASPAR TF sites.

**Table 5:** Representative biological processes and anatomic localizations within the two gene coexpression modules.

Module	Biological Process	Genes
**Collagen Module**	Collagen Catabolism	COL17A1; PRTN3; MMP8; MMP9; ELANE
	Cell surface interactions at the vascular wall	CEACAM1; CEACAM6; OLR1; CEACAM8
	Host responses to bacterial pathogens	CEBPE; ANXA3; DEFA4; HP; DEFA3; RNASE3; DEFA1; AZU1; BPI; PGLYRP1; CAMP; ELANE; LTF
	Mucosal innate immunity	DEFA3; RNASE3; DEFA1; CAMP; LTF
	**Anatomical localization**	**Genes**
	Extracellular space	SERPINB10; ORM1; ARG1; DEFA4; CRISP3; HP; DEFA3; DEFA1; RNASE3; MMP8; OLFM4; MMP9; MPO; TCN1; LCN2; CHI3L1; PRTN3; CTSG; CEACAM8; CAMP; LTF
	Extracellular matrix	SLPI; CRISP3; TFF3; CHI3L1; PRTN3; CTSG; DEFA1; MMP8; MMP9
**Platelet Module**	Transcription regulation	SCL; GATA1; HNF4A; FLI1; EGR1; SOX2; ATF3; RUNX1; GATA2; MITF; TAL1
	Coagulation and hemostasis	GUCY1B3; SPARC; ITGB3; PROS1; ITGA2B; MPL; F13A1; TFPI; THBS1; GRB14; HMG20B; JAM3; P2RY12; GUCY1A3; ABCC4; VWF; EGF; ACTN1; GP1BA; ANO6; PPBP; GP6; PTK2; TUBA4A; SELP; GP9; EHD3; KIF2A; MMRN1; CD9; PDE5A; ESAM; MGLL; VCL
	Platelet activation, aggregation, and signaling.	SPARC; ITGB3; PROS1; ITGA2B; MPL; F13A1; THBS1; GNG8; P2RY12; ABCC4; VWF; EGF; ACTN1; GP1BA; PPBP; GP6; GNG11; PTK2; TUBA4A; SELP; GP9; MMRN1; CD9; MGLL; VCL

### Bayesian Network Analysis

**Figure 3:**
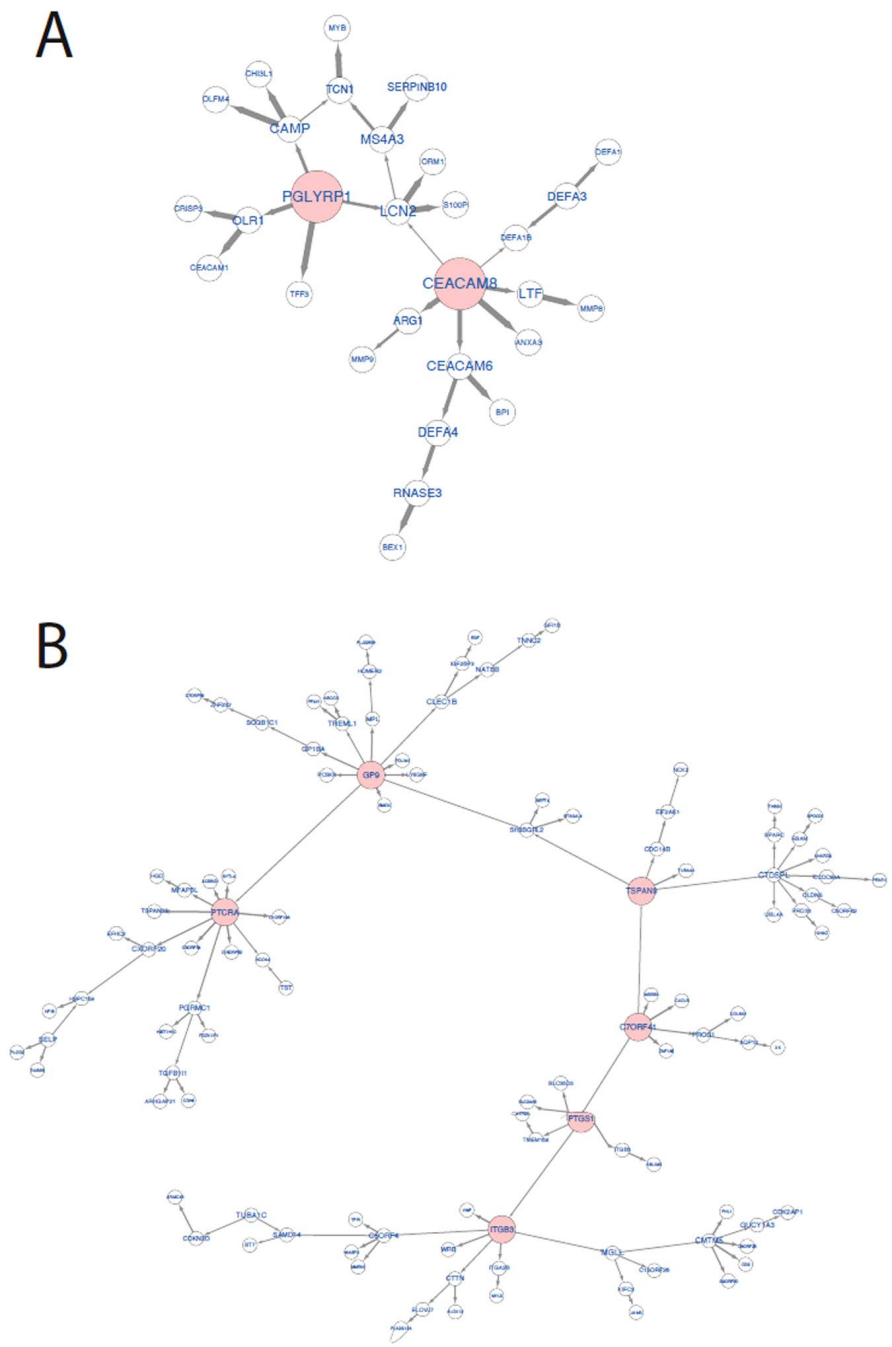
Bayesian network directed subgraphs around key driver genes.

We detected a total of 11 key driver (KD) genes within the two subgraphs of our significant WGCNA gene modules. The collagen module contained two KDs (*CEACAM8* and *PGLYRP1*) and the platelet module contained 9 KD genes (*GP9, C7orf41, HIST1H2AC, PTGS1, ITGB3, PTCRA, TSPAN9, TUBB1, NRGN*) (Fig. 3: Bayesian network directed subgraphs around key driver genes). *CEACAM8* (Carcinoembryonic Antigen Related Cell Adhesion Molecule 8) is a cell adhesion marker expressed on granulocytes involved in cellular adhesion to endothelial cells^19^. *PGLYRP1* (Peptidoglycan Recognition Protein 4) is a broadly bactericidal component of the innate immune system that encodes a receptor for bacterial cell wall peptidoglycans. This gene has been most studied in the context of immune-related disease such as dermatitis^20^. Both of these key driver genes are found in the extracellular space.

Several of the KD genes identified within the platelet module have well-characterized roles in platelet biology, cardiovascular physiology, and cardiovascular disease. *PTGS1* (prostaglandin-endoperoxide synthase 1, more commonly recognized clinically as COX-*1* or Cyclooxygenase 1) is one of two cyclooxygenase isoforms involved in the human biosynthesis of prostaglandins from arachidonic acid^21^. It is one of the two major targets of nonselective Non steroidal anti-inflammatory drug (NSAID) therapy. COX-1 is a vital regulator of angiogenesis in endothelial cells and has a role in platelet function via its control of downstream production of Thromboxane A_2_ (TXA_2_), a potent vasoconstrictor and prothrombotic agent released by activated platelets. *ITGB3*, better known clinically as Glycoprotein IIIa (GPIIIa) is a vital component of the GPIIb/IIIa integrin complex found on platelets and is a fibrinogen receptor. ADP activation of platelets leads to GPIIb/IIIa conformational changes, which induce binding to fibrinogen. This compound is the target of the antiplatelet drugs GPIIb/IIIa inhibitors used during percutaneous coronary intervention^22^. Dysfunction of ITGB3 is classically associated with Glanzmann Thrombasthenia^23^. *GP9* (Glycoprotein 9 or GPIX) is a platelet surface glycoprotein, which under shear stress binds GP1b and GPV to form a complex that acts as the von Willebrand Factor (vWF) receptor, and is thus a vital component of hemostasis. Mutations in the GPIIb/V/IX complex are classically associated with Bernard-Soulier syndrome^24^. *TUBB1* (Tubulin Beta 1 Class IV) is a component of one of the two families of tubulins that form microtubules. *TUBB1* is expressed in platelets and progenitor megakaryocytes and is intimately involved in platelet maturation. Shameer *et al*. demonstrated in a genome-phenome wide association study that 11 SNPs in *TUBB1* are associated with Mean platelet volume (MPV)^25^. *C7orf41* is an open reading frame on chromosome seven also known as Maturin (*MTURN)*. *C7orf41* is thought to be associated with neurogenesis, but it was recently shown to promote a novel pathway for megakaryocyte differentiation via ERK and JNK signaling pathways^26^. *NGRN* (Neurogranin) is another protein associated with neuronal physiology; specifically, it has been previously associated with Alzheimer Disease^27^. Although platelets are anucleate, they do contain a small amount of mRNA and maintain protein transcriptional activities. Transcriptional profiling of platelets demonstrates that *NGRN* is one of the top five most transcribed proteins within platelets^28^. *TSPAN9* (tetraspanin 9) is a poorly studied tetraspanin molecule specific to platelets and important for platelet function via interactions with GPVI^29^. The two remaining KD genes *PTCRA* (pre-T cell antigen receptor alpha) and *HIST1H2AC* (Histone H2AC, a member of one of the four core histones) do not have well-characterized roles in platelet function.

## Discussion

In this post-hoc analysis of the YELLOW II study, patients with DM showed a significant increase in FCT and improvement in CEC as seen in patients without DM, with comparable reductions in LDL-C. Despite these favorable reductions, the magnitude of reduction in inflammatory biomarkers and macrophage content was attenuated in the setting of DM. This could be explained by existence of both LDL-C dependent and LDL-C independent mechanisms of plaque regression induced by statins. Gene expression analysis suggests changes in pathways that relate to platelet activation and modulation of the fibrous cap beyond just thickness that might render patients with DM at higher residual cardiovascular risk despite achieving evidence based lipid goals.

Plaque composition plays a role in plaque disruption and thrombosis. Atherosclerotic lesions in patients with acute coronary syndromes and sudden cardiac death were found to have large lipid cores and increased macrophage infiltration leading to a higher risk for disruption than sclerotic plaques^30, 31^. In a postmortem study of patients who presented with sudden cardiac death, coronary plaques in diabetic patients were associated with larger necrotic core size and more diffuse atherosclerosis with inflammatory cell infiltrates, such as macrophages and T lymphocytes^32^. Inflammatory macrophages, often referred to as M1 macrophages, are involved in pathogen recognition and inflammatory cytokine secretion. Tissue repair is thought to be mediated by M2, or alternatively activated, macrophages^33^. Macrophages are also capable of degrading the extracellular matrix by phagocytosis or by secreting proteolytic enzymes—in particular, a family of metalloproteinases (MMPs) which may weaken the fibrous cap, predisposing its rupture^34^.

The inhibitory effect of statins on MMP-9 secretion suggests a potential stabilizing effect of these drugs on the atherosclerotic plaque^35^. Parathath *et al*. demonstrated in their mouse model that many of the benefits on plaque composition i.e. fewer M1 macrophages, more collagen, less inflammation, and more M2 macrophages after lipid reduction were attenuated in diabetic mice^36^. In our study, although there was a reduction in several macrophage parameters, we did show a residual increase in macrophage content. The difference in plaques seen in our study compared to the previous studies is that the lipid plaques were of patients with stable CAD and hence the inflammatory infiltrate at baseline may not have been different.

It is also known that systemic and coronary inflammation play an important role in the initiation, progression, and precipitation of atherothrombosis superimposed over traditional risk factors^37^.

Several markers of inflammation have been evaluated in the previous years. C-reactive protein (CRP), a non-specific marker of inflammation, has been proven to be one of the strongest predictors of the risk of cardiovascular events in patients with cardiovascular disease^38^. In patients with type 2 diabetes, CRP levels have also been shown to predict future cardiovascular events^39^. IL-6 is an important regulator of CRP expression in the liver, and is released in abundance into circulation during inflammatory processes. Studies have shown that CRP and IL-6 variations are significantly associated, however there is no significant reduction in plasma IL-6 levels after statin treatment^40^. This suggests that other cytokines involved in CRP regulation (TNF-α, interleukin 1β) may be the primary factors for statin effects. IL-1β^41^ and TNF-α are involved in the pathogenesis of DM and the oxidative stress caused by these pro-inflammatory cytokines in the setting of DM may blunt the effect of statins in regulating CRP levels.

Our transcriptomic findings support our vascular imaging findings. As this was a secondary, subgroup analysis, we can likely explain the paucity of genes differentially expressed between DM and non-DM patients as a result of limited power coupled with the relatively minor magnitude of expression changes that result from statin therapy, as demonstrated in our initial YELLOW II study. However, through our approach of combining two complementary forms of network inference, weighted gene Coexpression networks (WGCN), and Bayesian networks (BN), we were able to alleviate power concerns and detect biologically sensible and meaningful results.

The two modules of co-expressed genes correlate well with imaging findings. First, we demonstrated that our gene module that clinically correlated with FCT (Collagen Module) had significant roles in collagen and extracellular matrix synthesis and function. Collagen is an important component of the extracellular matrix of the arterial wall. Evidence suggests that the amount and organization of matrix collagen is associated with the mechanical stability of the fibrous cap. Smooth muscle cells (SMCs) in atherosclerotic plaques are responsible for synthesizing collagen. In the normal artery, SMCs are present in the media and perform a contractile role. In atherosclerotic plaques, SMCs migrate from the media to the intimal layer, resulting in an increase in collagen content^42^. Inflammatory cells such as macrophages in inflamed atheromas release matrix metalloproteinases (MMPs), causing the proteolysis of matrix collagen accompanied by the apoptosis of intimal SMCs, which impedes collagen synthesis^42^. This dynamic imbalance between collagen synthesis and degradation causes a net reduction in collagen content in the fibrous cap, which may predispose a plaque to rupture. Plaque stabilization with lipid-lowering therapy reverses this process, in both the cap and lipid pool, by restoring collagen production and reversing the effects of collagen degradation^43^. Plaques from subjects with DM are characterized by markedly reduced levels of collagen and elastin, connective tissue proteins that are critical in maintaining the stability of atherosclerotic plaques^30^. In our WGCN analysis we found differences in genes expressed for collagen catabolism between DM and non-DM patients, which could suggest the increase plaque vulnerability seen in DM patients. Interestingly, our WGCN analysis found that this module was also associated with innate immune responses to bacterial pathogens, which correlates well with the possible role of M1 macrophage function in relation to plaque composition. Our BN analysis confirmed the role of this module in immunity, as both identified key driver genes (*CEACAM8* and *PGLYRP1*) are involved in host responses to pathogens mediated by the innate immune system.

The second WGCNA gene module (platelet module) along with our identified key driver genes suggests that a key feature of diabetes is platelet dysfunction. Indeed, as vascular and platelet dysfunction is a precursor for coronary artery disease, this finding is sensible biologically. It has been known for some time that platelet dysfunction is integral to diabetic vasculopathy^44^. Studies have explored the effect that statins exert a direct antiplatelet effect independent of their cholesterol lowering properties. Diabetic patients are known to have a systemic isoprostane overproduction^45^, which are a family of eicosanoids derived from arachidonic acid interaction with reactive oxidant species (ROS). ROS generated by NOX2, play a crucial role in platelet isoprostane formation and serve to propagate platelet activation, amplifying platelet response to common agonists via glycoprotein (Gp)IIb/IIIa activation. Statins’ antiplatelet effects appear to occur by NOX2 downregulation, with ensuing platelet isoprostane lowering^46^. It is unknown whether this effect is blunted in DM patients given the overproduction of isoprostanes and the differential expression of genes regulating platelet activation. Our discovery that well-known mediators of platelet function such as COX-1, GPIIIa, and GPIX are implicated as key drivers of gene regulation in diabetic states confirms the plausibility of our biological findings. Generally, key driver nodes within gene regulatory networks are attractive targets for pharmacological intervention. Indeed, these genes are currently targeted by approved compounds such as NSAIDs and GPIIb/IIIa inhibitors like abciximab. However, as there is only emerging evidence for the roles of identified genes such as *MTURN*, *NGRN*, and *TSPAN9* in platelet function, we believe that these may ultimately prove to be attractive and novel targets for future drug development.

## Limitations

The duration of follow up was short and a longer follow up time may demonstrate morphological differences between the two groups. As this was a secondary, subgroup analysis, there was a paucity of genes differentially expressed between DM and non-DM patients due to limited power. We note that the gene expression findings we observed may have a genetic basis which we could not assay in this study. Due to the previously demonstrated benefits of intensive statin therapy for secondary prevention of cardiovascular disease, we could not ethically randomize patients to a placebo control arm.

## Conclusion

The study findings suggest that aggressive statin therapy achieves significant reduction in LDL-C levels and stabilizes the plaques in diabetics as in non-diabetics. However, the genetic results suggest changes in diabetics related to platelets and properties of the fibrous cap other than thickness that might render patients with DM at a higher residual cardiovascular risk despite goal lowering LDL-C levels. We believe these findings may present a step towards better understanding of diabetes specific cardiovascular pathology and may ultimately help to guide future therapy development.

## Methods

### Study Design and Procedures

The design of the YELLOW II study has been previously described^15^. From August 2013 to February 2015, 91 patients with multivessel CAD were enrolled in the original YELLOW II study. Eighty five patients underwent PCI for a culprit lesion followed by OCT and NIRS/IVUS imaging of an obstructive NCL. Patients were categorized by presence or absence of DM at baseline (Supplementary Figure 2). DM was defined according to clinical history and/or use of oral hypoglycemic agents or insulin. Following enrollment, all subjects received 40 mg of rosuvastatin daily. At 8–12 weeks post-enrollment (follow-up), the NCL was reimaged as a part of staged intervention. All procedures were performed at the Mount Sinai Catheterization laboratory. Serial changes of serum lipids, high-sensitivity C-reactive protein (hs-CRP), and apolipoproteins were assessed at baseline and follow-up procedures. In addition, fasting blood samples were obtained during both baseline and follow-up for CEC quantification and PBMC isolation. Off-line gray-scale IVUS, NIRS and OCT analyses were performed by an independent laboratory, the Cardiovascular Research Foundation (New York, NY), according to the current guidelines^47, 48^. Statistical analyses were performed by the data-coordinating center at the Cardiovascular Institute of Mount Sinai Hospital (New York, New York).

### Statistical Analysis

Categorical variables are presented as counts and percentage and compared using a chi-square or Fisher’s exact test. Continuous measurements are expressed as mean ± SD for normally distributed variables or median and interquartile range (25th percentile, 75th percentile) for nonparametric data. The Shapiro-Wilk test was used to assess the normality of continuous data. The significance of continuous variables at follow-up compared to baseline measurements was assessed using the Wilcoxon signed-rank test. The Student t-test or Mann-Whitney U test was used for between-group comparisons of biochemical parameters and plaque characteristics. The biochemical parameters and CEC after therapy were calculated by subtracting the baseline values from those at follow up. In addition, we performed multivariate linear regression analysis to examine whether DM has an independent effect on the changes in CEC and plaque morphology after statin treatment. DM status represented the exposure of interest in each statistical model, while dependent outcomes included different measures of changes in plaque severity assessed by OCT (fibrous cap thickness, lipid arc and length, lipid volume index, macrophage arc and length), IVUS (plaque burden, percent atheroma volume and total atheroma volume), and NIRS (maxLCBI4mm). Variables demonstrating significant differences between patients with and without DM were included as additional covariates of DM in each model in addition to known risk factors (smoking, hypertension, BMI). Results were reported as beta coefficients with 95% confidence intervals (CIs) and p values. All reported P values are two-tailed, with a P value < 0.05 indicating statistical significance. All analyses were performed with the use of SAS software, version 9.4.

### Translational Studies

Cholesterol efflux capacity (CEC) was quantified by measuring the efflux of radiolabeled cholesterol from mouse cell line J774 macrophages to patient apolipoprotein B (apoB)-depleted serum as previously described^49, 50^. All the assays were performed in triplicate.

### Microarray Preparation and Differential Gene Expression Analysis

PBMCs were isolated from patient blood by density gradient centrifugation using Ficollpaque premium. RNA was isolated using TRIzol protocol and gene expression profiling was performed using the Illumina Human HT-12 v4 bead chip array as previously described^15^. We performed quality control, variance-stabilizing transformation normalization, filtering of unexpressed transcripts, and correction for any batch effects resulting from preparation of the microarrays as previously described. Gene expression data is available from the NIH Gene Expression Omnibus (GEO) website with the ID GSE86216. We fit mixed linear models to the processed probe expression values and evaluated regression model coefficients with the empirical Bayes method. The model was parameterized using a 2 × 2 factorial design in which we estimated the following contrasts: (1) Differences in DM patients from baseline to follow-up; (2) differences in non-DM patients from baseline to follow-up; (3) differences between DM and non-DM patients, controlling for changes from baseline to follow-up (interaction term). All regression coefficient P values were adjusted for multiple testing with the Benjamini-Hochberg (BH) method to control for false discovery at a 5% rate (5% FDR).

### Weighted Gene Coexpression Analysis

We used weighted gene coexpression analysis (WGCNA)^51^ to discover clusters (or “modules”) of co-expressed genes. Genes are coexpressed if their expression values are correlated with each other. Briefly, in WGCNA genes are considered as nodes in a network whose adjacency is weighted by their coexpression on a logarithmic scale. We then apply hierarchical clustering on the constructed network to identify gene modules, where branches of the constructed dendrogram correspond to unique gene modules. We tested the first principal component of each gene module’s expression matrix (called an “eigengene”) for correlation with DM status. Eigengenes can be considered a vector representing the weighted expression profile of all module genes^51^. Gene sets from the two modules whose eigengenes were associated with diabetes status were then queried with EnrichR^52^ to test for the statistical enrichment of gene functional and ontological properties from several databases. As above, we corrected reported P values at a 5% FDR with the BH method. We conducted a simulation study to analyze the reproducibility of gene expression modules (Supplementary Figure 3).

### Bayesian Network Generation

We used the software RIMBAnet to construct Bayesian networks (BNs) from gene expression data^53^. The continuous gene expression data was discretized into three states for each gene: high expression, low expression, and unexpressed. States were assigned by first normalizing each gene’s expression values across individuals and then K-means clustering (k = 3) was performed with the option of reclustering if the gene did not have an even distribution across the original three clusters, in which case, the gene would be classified into two clusters: high and low expression. The maximum number of parent nodes allowed for any given node was set to 3. After successfully running 1,000 reconstructions, we pooled the networks together to create a consensus network. BNs are directed acyclic graphs (DAG) by definition; therefore, in the event of a cycle in the consensus network, the edge present the least number of times in 1,000 reconstructions was removed to break the cycle. This process was repeated until no cycles were present and the resulting network was a DAG.

### Identification of the largest subgraph and key driver nodes

Nodes from relevant coexpression modules were projected on the BN generated from blood samples collected at the follow-up visit. The largest subgraph was constructed by selecting the first-degree neighbors of nodes in either direction (in-degree or out-degree) projected onto the BN to identify the most highly connected sub-structure. Key driver nodes (KDs) were detected by calculating the shortest downstream path length between each pair of nodes in the network. For each candidate key driver node, we calculated the inverse of path length between the candidate key driver node and every other node in the network. The inverse path lengths were then summed to obtain a final connectivity score per node. Based on this calculation, nodes in the 97.5th percentile of highest connectivity were classified as KDs.

### Data Availability Statement

Gene expression data was submitted to Gene Expression Omnibus (GEO) database, the series accession ID GSE86216.

## Electronic supplementary material

Supplementary Information
