# Supplementary material for: Intracoronary Imaging, Cholesterol Efflux, and Transcriptomics after Intensive Statin Treatment in Diabetes
Source: Sci Rep. 2017 Aug 1;7:7001. doi: 10.1038/s41598-017-07029-7 (PMC5539108; doi:10.1038/s41598-017-07029-7)

SUPPLEMENTARY INFORMATION

Intracoronary Imaging, Cholesterol Efflux, and Transcriptomics after Intensive Statin Treatment in Diabetes

Surbhi Chamaria[1], Kipp W. Johnson[2,3] , Yuliya Vengrenyuk[1], Usman Baber[1], Shameer Khader[2,3], Aparna Divaraniya[2,3], Benjamin S. Glicksberg[2,3], Li Li[2,3], Samit Bhatheja[1], Pedro Moreno[1], Akiko Maehara[5], Roxana Mehran[1], Joel T. Dudley[2,3,4], Jagat Narula[1], Samin K. Sharma[1], Annapoorna S. Kini[1]

Affiliations

1. Mount Sinai Heart, Mount Sinai Hospital, New York, USA
2. Institute for Next Generation Healthcare, Icahn School of Medicine at Mount Sinai, New York, USA
3. Department of Genetics and Genomic Sciences, Icahn Institute for Genetics and Genomic Sciences, Icahn School of Medicine, New York, USA
4. Department of Population Health and Health Policy, Icahn School of Medicine at Mount Sinai, New York, USA
5. Columbia University Medical Center, New York, USA

Supplementary Table 1: Types of Anti-diabetic medications according to medication class

Diabetes Medications	No.
**Sulfonylureas**	
Glyburide	4
Glipizide	9
Glimepiride	3
**Metformin**	27
**Other Oral Hypoglycemics**	
Sitagliptin	8
Pioglitazone	2
Repaglinide	1
Saxagliptine	1
Linagliptin	1
Acarbose	1
**Insulin**	10

Supplementary Table 2: Multivariate regression analysis of the association between DM and changes in plaque characteristics

Model number	Change in plaque characteristic	Standardized coefficient (95% CI)	P-value
**OCT**			
1	ΔFCT (min)	-0.14(-13.36 to 3.72)	0.26
2	ΔLA (max)	-0.22(-28.2 to 0.38)	0.06
3	ΔLL	-0.6(-0.63 to 0.38)	0.62
4	ΔLVI	0.12(-79.8 to 263.6)	0.29
5	ΔMA (max)	-0.12(-26.2 to 8.7)	0.32
6	ΔML	-0.24(-2.6 to 0.11)	0.07
**IVUS**			
7	ΔPB	0.01(-2.2 to 2.3)	0.95
8	ΔPAV	-0.01(-1.1 to 0.99)	0.92
9	ΔTAV	0.12(-3.2 to 10.7)	0.29
**NIRS**			
10	ΔmaxLCBI4mm	-0.21(-132.3 to 1.24)	0.05

OCT Optical Coherence Tomography, FCT fibrous cap thickness, LA lipid arc, LL lipid length, LVI lipid volume index, MA macrophage arc, ML macrophage length, IVUS intravascular ultrasound, PB plaque burden, PAV percent atheroma volume, TAV total atheroma volume, maxLCBI4mm maximal lipid core burden index in a 4 mm segment

Supplementary Figure 1: Differential expression heatmap

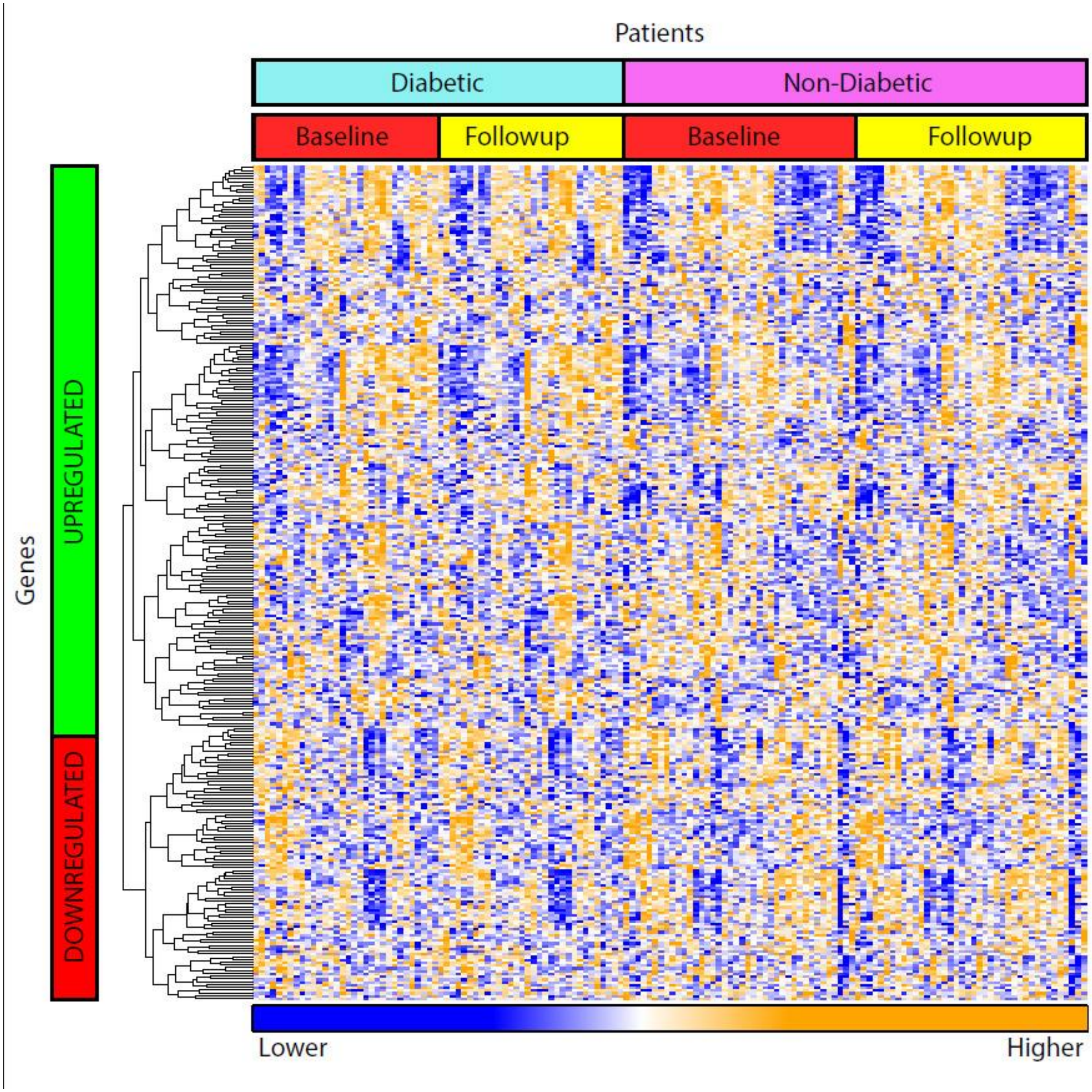

Supplementary Figure 2: Flow diagram for patient enrollment in the substudy

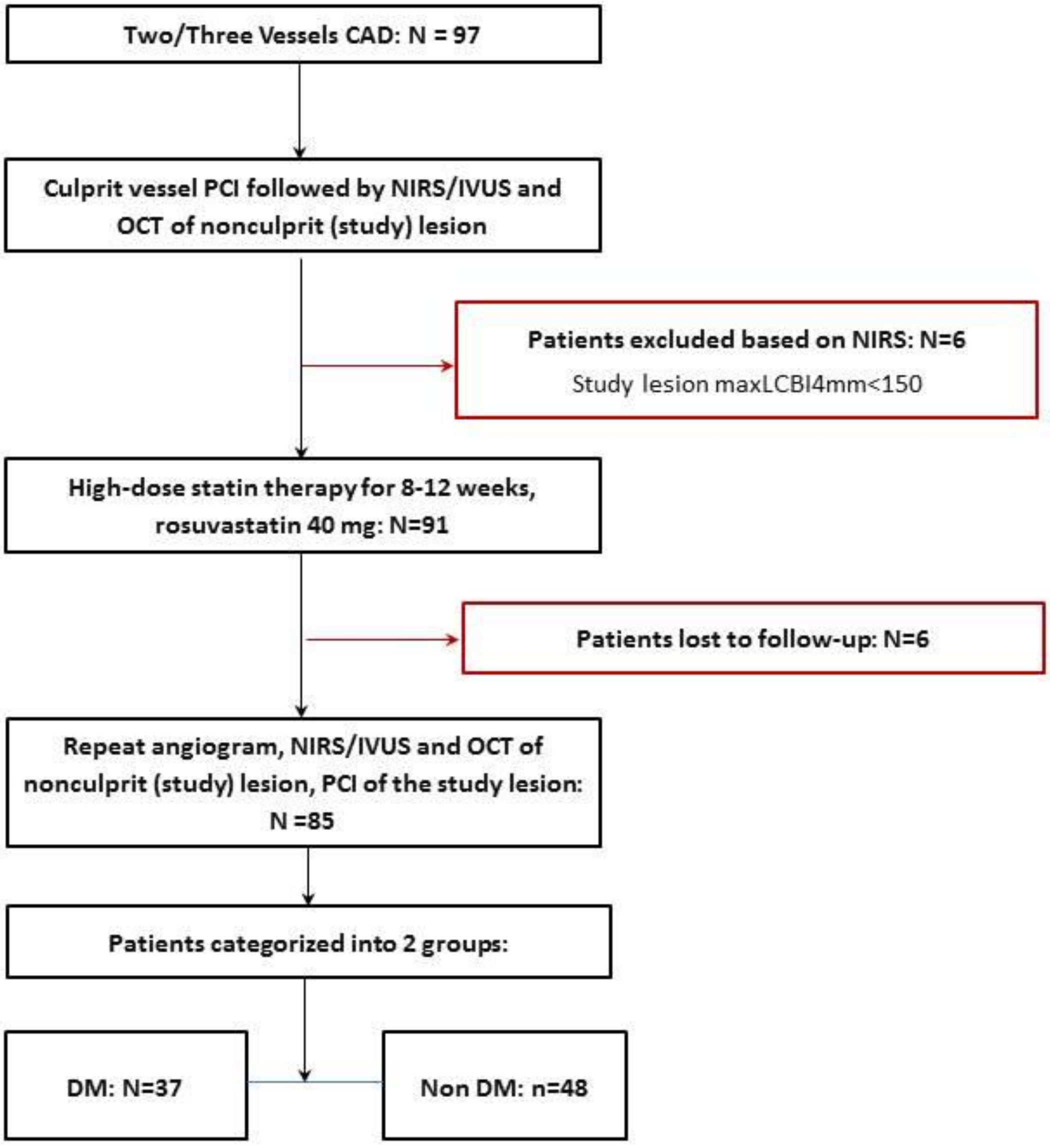

CAD – coronary artery disease, PCI- percutaneous coronary intervention, NIRS- near infrared spectroscopy, IVUS- intravascular ultrasound, OCT- optical coherence tomography, maxLCBI4mm maximal lipid core burden index in a 4mm segment, DM- Diabetes Mellitus

Supplementary Figure 3: WGCNA Network Preservation Simulation Study

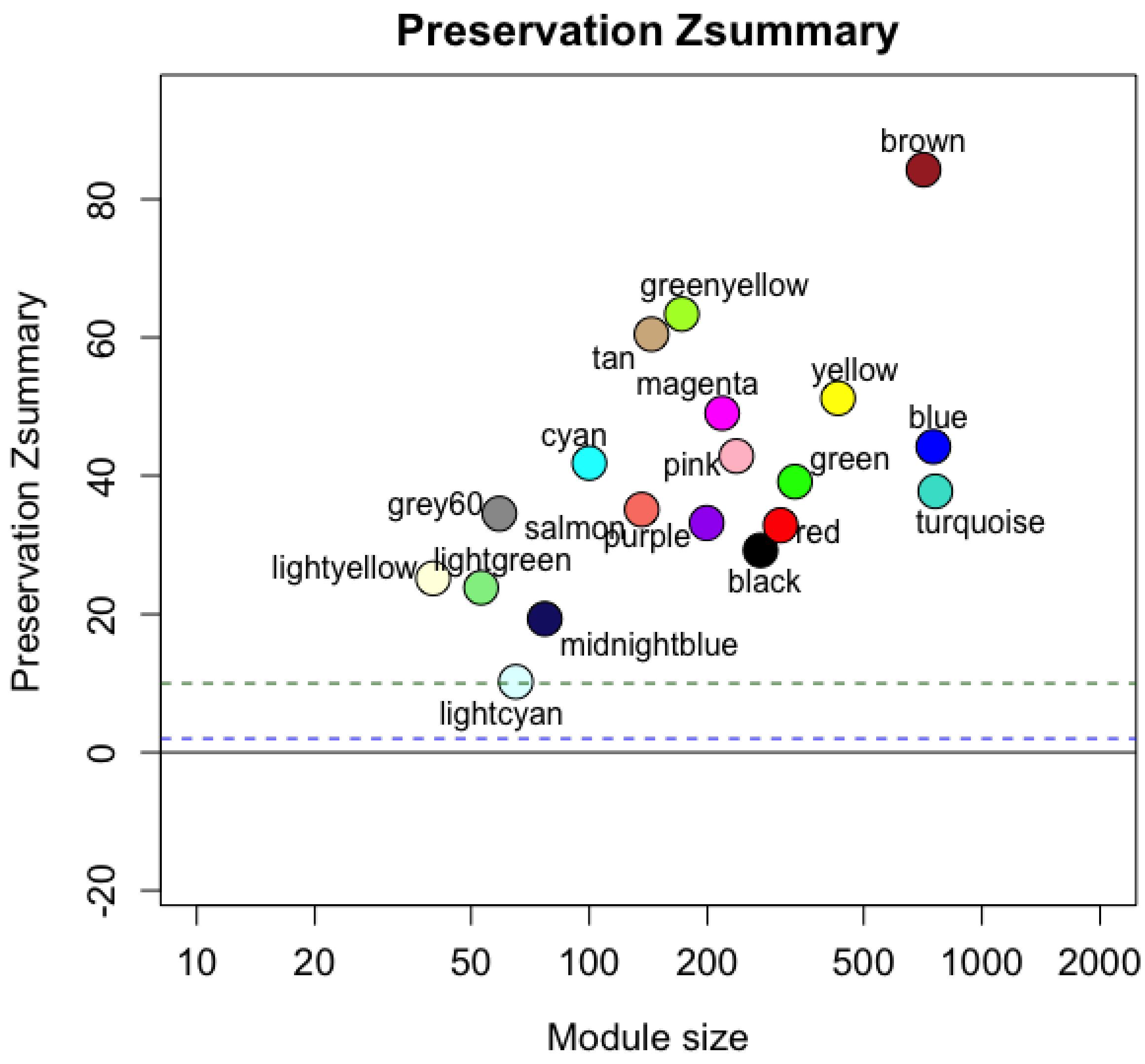

Supplement: Supplementary file 1 — Supplementary Information [file 41598_2017_7029_MOESM1_ESM.pdf]
